# Skin aging from mechanisms to interventions: focusing on dermal aging

**DOI:** 10.3389/fphys.2023.1195272

**Published:** 2023-05-10

**Authors:** Sun Hye Shin, Yoon Hwan Lee, Nark-Kyoung Rho, Kui Young Park

**Affiliations:** ^1^ Department of Dermatology, Chung-Ang University College of Medicine, Seoul, Republic of Korea; ^2^ Leaders Aesthetic Laser & Cosmetic Surgery Center, Seoul, Republic of Korea

**Keywords:** cellular senescence, dermal fibroblast, rejuvenation, skin aging, senotherapeutics

## Abstract

Skin aging is a multifaceted process that involves intrinsic and extrinsic mechanisms that lead to various structural and physiological changes in the skin. Intrinsic aging is associated with programmed aging and cellular senescence, which are caused by endogenous oxidative stress and cellular damage. Extrinsic aging is the result of environmental factors, such as ultraviolet (UV) radiation and pollution, and leads to the production of reactive oxygen species, ultimately causing DNA damage and cellular dysfunction. In aged skin, senescent cells accumulate and contribute to the degradation of the extracellular matrix, which further contributes to the aging process. To combat the symptoms of aging, various topical agents and clinical procedures such as chemical peels, injectables, and energy-based devices have been developed. These procedures address different symptoms of aging, but to devise an effective anti-aging treatment protocol, it is essential to thoroughly understand the mechanisms of skin aging. This review provides an overview of the mechanisms of skin aging and their significance in the development of anti-aging treatments.

## 1 Introduction

Skin aging is a complex process that involves numerous biological and biochemical changes as well as secondary structural changes of the skin, underlying muscles, subcutaneous fat tissue, and bony structures. Common aesthetic procedures performed in clinical practice, such as chemical peels, energy-based treatments, injectable treatments, and threads, may share similar mechanisms; however, they often address the symptoms and signs of skin aging in distinct ways. Furthermore, as research on the mechanism of skin aging continues to expand, existing theories are replaced with new concepts, such as cellular senescence of dermal fibroblasts (Fang et al., 2022; Shvedova et al., 2022). Thus, clinicians must possess a thorough comprehension of skin aging physiology to devise a treatment plan that entails selecting anti-aging procedures that target specific mechanisms of skin aging while simultaneously reducing side effects. It is hoped that this narrative review will provide new avenues to comprehensively describe the complex skin aging process and help clinicians to establish anti-aging treatment protocols.

## 2 Mechanisms of skin aging

### 2.1 Molecular mechanisms of skin aging

The skin is the body’s largest organ and is continuously exposed to various environmental factors, including ultraviolet (UV) rays, smoking, heat, and air pollution. Therefore, the skin undergoes extrinsic aging as well as intrinsic aging, which is also referred to as chronological aging. The process of intrinsic aging can be considered alongside programmed aging, and it results from continuous chromatic damage by various factors, of which the most representative is oxidative stress caused by reactive oxygen species (ROS). The cells have an endogenous defense system against oxidative stress including superoxide dismutase (SOD), tripeptide glutathione, and catalase (Steenvoorden and van Henegouwen, 1997). Age-related impairment in its redox capacity results in the accumulation of ROS, thereby causing a detrimental effect on cellular components including proteins, lipids, and DNA, consequently leading to cellular dysfunction (Gniadecka et al., 1998; Gu et al., 2020). ROS generated by exogenous factors such as UV rays and air pollution also play a significant role in extrinsic aging.

In response to stress factors including DNA damage, cells enter a state of irreversible growth arrest, which is called cellular senescence (Hayflick, 1965). Recent research has uncovered that cellular senescence plays a major role in the skin aging process (Fitsiou et al., 2021; Wlaschek et al., 2021; Kim et al., 2022a; Kim et al., 2022b; Gerasymchuk et al., 2022; Papaccio et al., 2022). Senescent cells exhibit several biomarkers: 1) increased activity of the cell cycle arrest proteins p21WAF1 and p16INK4A, 2) lysosomal enzyme senescence-associated β galactosidase (SA-β-gal), and 3) decreased expression of nuclear high mobility group box-1 (HMGB1) and lamin B1, a structural component of the nuclear lamina (Ho and Dreesen, 2021). They also release humoral factors known as senescence-associated secretory phenotype (SASP), which includes various inflammatory cytokines, chemokines, matrix proteases, and microRNAs (Coppe et al., 2010; Kim et al., 2016). The temporary cellular senescence signals that physiologically occur during wound healing promote the formation of granulation tissue and skin regeneration while inhibiting excessive cell growth that can progress to precancerous or cancerous lesions (Demaria et al., 2014; Wang and Dreesen, 2018). As age increases, the accumulation of senescent keratinocytes, melanocytes, and, most importantly, fibroblasts can cause various age-related diseases and disrupt the homeostasis of the skin (Wlaschek et al., 2021).

Moreover, the degradation of the extracellular matrix (ECM) is observed as a result of altered senescent cells and excessive ROS production. Excessive ROS activate the mitogen-activated protein kinase (MAPK)/activator protein 1 (AP-1) pathway, which consequently induces the expression of matrix metalloproteinase (MMP), resulting in collagen breakdown (Chung et al., 2000). It also downregulates the collagen production via the TGF-β/Smad signaling pathway (Quan et al., 2004; Quan et al., 2010; He et al., 2014). In addition, tissue inhibitors of metalloproteinases (TIMPs) are downregulated during the aging process. Furthermore, the presence of senescent cells contributes to ECM degradation by promoting chronic inflammatory responses and collagen breakdown. In particular, the senescent fibroblasts express SASP containing MMP-2, MMP-9, and proinflammatory cytokines such as interleukin (IL)-6 and IL-8 (Kuilman et al., 2008; Wang and Dreesen, 2018; Wlaschek et al., 2021). The migration of neutrophils after inflammation or UV exposure further accelerates the collagen and elastin fragmentation via production of neutrophil-derived proteolytic enzymes (Li et al., 2013; Sharma et al., 2020).

### 2.2 Dermal aging

These two types of skin aging have several overlapping molecular mechanisms including ROS generation, DNA damage, and structural deterioration of ECM components. Therefore, the clinical phenotypes of skin aging are similar in some respects; however, some facets differ based on the aging process (Park, 2022). Intrinsic aging results in overall thinning of the skin, dry and pale skin, fine wrinkles, and skin sagging with decreased elasticity (Walker, 2022). The functions of the sweat and sebaceous glands also decrease, with less sebum secretion caused by decreased peroxisome proliferator-activated receptor gamma (PPAR-γ) expression ultimately leading to dry skin, while sebaceous gland hyperplasia can occur due to increased gland size (Zouboulis and Boschnakow, 2001; Kim et al., 2014). Furthermore, extrinsic aging manifests as relatively coarse wrinkles, severe loss of elasticity, and dyspigmentation (Walker, 2022).

Despite these differences, biochemical and biophysical changes in the dermis are common to both aging processes and are major contributing factors to the aging phenotypes such as wrinkles and loss of skin elasticity. The dermis of the skin consists of connective tissue that is rich in collagen, which provides mechanical support and structure. Recently, the changes in the dermal components in skin aging and treatments to reverse or combat them to reduce the signs of aging have become the focus of many dermatologists. Therefore, in this part, we will discuss these dermal aging processes in more detail.

The fibroblasts within the dermis are responsible for the synthesis, organization, and remodeling of collagen and thus play a major role in maintaining the integrity of the ECM. As aforementioned, aging causes the accumulation of senescent fibroblasts in the dermis, which causes gradual degradation and dysfunction of the ECM via release of proteolytic, matrix-degrading SASPs (Ressler et al., 2006).

In particular, the matricellular protein CCN1, also known as cysteine-rich protein 61, has been suggested to be a contributor to the age-associated dermal microenvironment. CCN1 is markedly elevated in the human dermal fibroblasts in aged skin, and Quan et al. demonstrated that elevated expression of CCN1 accelerates dermal aging by dysregulating the production and homeostasis of collagen using a transgenic mouse model (Quan et al., 2011; Quan et al., 2021). Their results showed that the fibroblasts of COL1A2-CCN1 mice had increased MMP expression and impaired TGF-β/Smad signaling, resulting in reduced COL-1 expression and fragmentation of ECM. Furthermore, CCN1 induces increased expression of proinflammatory cytokines, thus further promoting dermal aging (Quan et al., 2011). In addition, Ezure et al. found that complement factor D secreted from senescent dermal fibroblasts induces increased MMP-1 expression and negatively impacts matrix production in surrounding young dermal fibroblasts *in vitro* (Ezure et al., 2019). Collectively, these changes disrupt the complex interaction of dermal fibroblasts with the ECM, including by reducing the mechanical forces exerted on the fibroblasts, which negatively affects their morphology and function (Qin et al., 2014; Fisher et al., 2016).

In addition, Solé-Boldo et al. (2020) found that there is decrease in the number and heterogeneity of dermal fibroblasts with age. These skin aging-associated changes were mainly observed in the papillary dermis rather than in the reticular dermis with decreased papillary dermal fibroblasts (Mine et al., 2008). Collectively, these changes impair the structure and function of the skin and create a microenvironment that is conducive to age-related skin pathologies, including delayed wound healing and skin cancer (Woodley, 2017; Blair et al., 2020; Fane and Weeraratna, 2020; Xue et al., 2022).

Furthermore, senescent fibroblasts contribute to skin aging by interacting with other neighboring cells, including keratinocytes and melanocytes, through paracrine signaling. Insulin-like growth factor (IGF)-1, which is mainly released by dermal fibroblasts, is known to be necessary for mesenchymal stem cell niches and the modulation of epidermal cell proliferation and differentiation (Hodak et al., 1996; Youssef et al., 2017; Muraguchi et al., 2019). In addition, IGF-1 signaling is essential for the appropriate protective responses (DNA damage response, DDR) of keratinocytes to UV-induced DNA damage by inducing favorable cellular senescence or DNA damage repair (Lewis et al., 2009; Loesch et al., 2016; Alkawar et al., 2020). Aged skin exhibits decreased synthesis of IGF-1, which results in the epidermal atrophy and proliferation of keratinocytes with unrepaired DNA, leading to the development of age-related non-melanoma skin cancers (Stachelscheid et al., 2008; Lewis et al., 2009; Alkawar et al., 2020; Wlaschek et al., 2021). More recently, Terlecki-Zaniewicz et al. (2019) showed that extracellular vesicles derived from the senescent fibroblasts affect the terminal differentiation of keratinocytes with decreased expression levels of involucrin in a 2D cell culture model, which is reported to be a major initiator of cornification. Senescent fibroblasts have also been suggested to contribute to age-related pigmentation by inducing activation of melanocytes through several factors such as secreted frizzled-related protein 2, growth differentiation factor 15, and stromal-derived factor 1 (Kim et al., 2016; Yoon et al., 2018; Kim Y. et al., 2020; Kim J. C. et al., 2022). This was also supported by reduced epidermal pigmentation after radiofrequency treatment with reduced p16INK4A-positive senescent fibroblasts in a pilot study by (Kim et al., 2019).

There are also age-related structural changes in the dermal elastic fibers. Elastic fiber networks are composed of elastin and fibrillin, forming a unique arrangement within the dermis. In the upper papillary dermis, oxytalan fibers, which are microfibrillar bundles abundant in fibrillin, play a role in preventing the epidermis from easily detaching from the dermo-epidermal junction (DEJ) by forming a candlestick-shaped organic bond with the DEJ (Heinz, 2021). In photoaged skin, the oxytalan fibers undergo degeneration, and the elastic fibers of upper dermis are degraded by elastolytic enzymes including MMPs and neutrophil elastases (Bernstein et al., 1996; Naylor et al., 2011; Bonta et al., 2013). In addition, the altered, disorganized elastic fibers gradually accumulate in the reticular dermis, appearing as solar elastosis. In contrast, intrinsic skin aging is characterized by overall depletion of the elastic fiber network (El-Domyati et al., 2002).

Recently, it has been recognized that the basement membrane not only provides physical support for keratinocytes but also plays a major role in the regulation of signaling and communication between epidermal and dermal cells (Tsutsui et al., 2021). With age, the protein components of the basement membrane zone, including collagen 7 and 17, nidogen, integrins, and laminin 332, decrease, and the papillary pattern of the DEJ flattens (Iriyama et al., 2011a; Amano, 2016; Roig-Rosello and Rousselle, 2020). It has been postulated that disrupted basement membranes allow soluble melanogenic regulators from senescent fibroblasts to more easily stimulate melanocyte activity and accelerate age-related pigmentation (Goyarts et al., 2007; Amano, 2009; Iriyama et al., 2011b; Bastonini et al., 2016). Iriyama et al. (2022) have shown that the inhibition of basement membrane degradation with MMP inhibitors and heparinase inhibitors promotes the deposition of laminin-511 at the DEJ, which in turn promotes the secretion of platelet-derived growth factor consisting of 2 B subunits (PDGF-BB). Expression of COL5A1 and COL1A1 genes was increased in the fibroblasts stimulated with PDGF-BB, suggesting increased collagen expression in the papillary dermis (Iriyama et al., 2022). Therefore, strengthening the damaged basement membrane and restoring epidermal-dermal integrity have been proposed as new anti-ageing targets, but the actual clinical significance of the DEJ and its role in aging requires much further research.

Age-related changes in proteoglycans (PGs) and glycosaminoglycans (GAGs) are very complex, and there are still many unknown aspects (Oh et al., 2011b; Lee et al., 2016). Although previous research has often reported conflicting results in the changes of PGs and GAGs, they have received attention as promising targets for skin rejuvenation (Oh et al., 2011a; Oh et al., 2011b; Lee et al., 2016; Wang et al., 2021). Unlike collagen, which has a relatively long half-life, GAGs, such as hyaluronic acid (HA), have a much shorter half-life ranging from 24 to 36 h in human skin (Jiang et al., 2007; Fallacara et al., 2018). While the regulation of collagen metabolism takes a long time to show any visible changes, GAGs have the advantage that a treatment effect can be observed within a short period of time. However, further follow-up studies are needed to understand the role of PGs and GAGs in skin aging.

Table 1 summarizes the differences between intrinsic and extrinsic aging of the skin that have been generally recognized to date. However, recent research suggests that this distinction is not as clear-cut as textbooks describe and can often be confusing. It would be more clinically appropriate to understand that middle-aged adults visiting dermatologic clinics for skin rejuvenation undergo concomitant intrinsic and extrinsic aging. Thus, the histological and molecular changes related to skin aging that have been acknowledged to date should be organized to establish an appropriate treatment plan.

**TABLE 1:** Histologic and biochemical differences between intrinsic and extrinsic aging.

Components	Intrinsic aging	Extrinsic aging	References
Epidermal HA	↓	↓ or ↔	Oh et al. (2011)
			Lee et al. (2016)
Dermal HA	↔	↑	Oh et al. (2011)
	Reduced extractability	Shortened length	Lee et al. (2016)
Dermal sGAGs	↓	↑	Oh et al. (2011)
		Clumped	Lee et al. (2016)
Proteins	Slightly altered structures	Markedly altered structures, Hydrophobic	Gniadecka et al. (1998)
Collagen fibers			Quan et al. (2004)
	↓	↑ or ↔	Li et al. (2013)
	Thinned, less soluble	Thickened, more soluble	Sharma et al. (2020)
	Fragmented, disorganized fibers		Chung et al. (2000)
	Decreased neocollagenesis, Type III to I ratio ↑		Wlaschek et al. (2021)
Elastic fibers	↓	↑ (Accumulation of altered fibers)	Bernstein et al. (1996)
			El-Domyati et al. (2002)
			Naylor et al. (2011)
			Bonta et al. (2013)

Abbreviations: GAG, glycosaminoglycan; HA, hyaluronic acid; sGAG, sulfated glycosaminoglycan.

## 3 Management of skin aging: focus on dermal aging

### 3.1 UV protection

As mentioned above, UV rays play a critical role in cellular aging and skin aging; thus, Sun protection—using sunscreen or protective clothing and staying in the shade—is the most basic and essential option for preventing skin aging and slowing the rate of aging-related changes.

### 3.2 Energy-based devices

Various energy-based devices, such as lasers, high-intensity focused ultrasound (HFU), and radiofrequency (RF) devices, have grown increasingly common to address aging phenotypes. These devices deliver thermal energy to the reticular dermis and subcutaneous tissue, which subsequently causes tissue contraction and stimulates neocollagenesis, leading to improvement in skin laxity and rhytides (Orringer et al., 2012; Majidian et al., 2021; Chen et al., 2022).

An ablative laser, such as a CO_2_ laser or an Erbium:YAG laser, which requires re-epithelialization, has been used in the past, but recently, a non-ablative fractional laser has been used mainly to reduce the downtime and risk of adverse events including postinflammatory hyperpigmentation or scarring (Nanni and Alster, 1998; Chen et al., 2022). In contrast, fractional picosecond lasers produce nonthermal, photomechanical stress in the dermis and promote fibroblast proliferation (Tanghetti, 2016; K et al., 2021). Recent *ex vivo* animal and clinical studies also support that 532-nm and 1,064-nm picosecond Nd:YAG lasers may improve photoaged skin (Yim et al., 2020; Connor et al., 2021; Han et al., 2023). In addition, various lasers including low fluence Q-switched Nd:YAG lasers, Q-switched ruby lasers, and Q-switched alexandrite lasers are effective for treating aging-related pigmentation through selective photothermolysis of melanosomes (Anderson and Parrish, 1983; Sadighha et al., 2008; Vachiramon et al., 2016).

HFU is a noninvasive and safe treatment that focuses ultrasound waves on a localized area, much like a magnifying glass focuses light, causing thermal coagulation of the subcutaneous tissue and rearranging the collagen and elastic fibers of the subcutaneous tissue without affecting the skin surface. In contrast, RF devices deliver relatively diffuse thermal energy throughout the dermis (Suh et al., 2015). Kwon et al. (2021) showed that bipolar RF device treatment reduces the number of p16INK4A-positive senescent fibroblasts and increases the expression of HSP70 and HSP90 in melasma skin. More recently, fractional RF microneedling devices that deliver targeted bipolar RF energy directly to the reticular dermis via microneedles have been developed. Fractional RF microneedling devices have also shown to be effective in treating UV-induced hyperpigmentation by upregulating the anti-senescence pathways (Rangarajan et al., 2013; Yoon et al., 2018; Lee et al., 2021).

Furthermore, recent evidence suggests that a light emitting diode (LED) can also ameliorate UV-induced changes in dermal fibroblasts and promote collagen synthesis by photobiomodulation (Baez and Reilly, 2007; Kim et al., 2015; Mamalis and Jagdeo, 2018; Hong et al., 2022). The mechanisms underlying the effects of LEDs aren’t fully understood, and clinical data are insufficient; therefore, further studies are needed.

### 3.3 Topical agents

A variety of topical agents have been used to improve the signs of skin aging, but retinoids are currently considered the most effective option (Samuel et al., 2005). Retinoids have been shown to increase types Ⅰ, III, and VII collagen and GAG deposition and to normalize elastic tissue organization (Woodley et al., 1990). In addition, topical tretinoin treatment also induces thickening of the granular layer and compaction of the stratum corneum, resulting in smooth skin (Berardesca et al., 1990). Clinical evidence also supports the role of topical retinoids in the reversal of skin aging phenotypes including fine wrinkling, dyschromia, and skin elasticity (Weinstein et al., 1991; Olsen et al., 1992; Darlenski et al., 2010; Milosheska and Roskar, 2022). Topical antioxidants, such as ascorbic acid (vitamin C), have also been shown to be effective in reducing skin aging. Ascorbic acid reduces ROS and is required for collagen synthesis in human skin fibroblasts. However, its poor skin penetration and chemical instability can reduce its clinical efficacy. In addition, chemical peeling using topical alpha-hydroxy acids, such as glycolic or lactic acid, have been shown to improve the quality of elastic fibers, stimulate GAG and collagen production in the dermis, and increase the epidermal thickness (Bernstein et al., 2001; Hussein et al., 2008). Tricholoroacetic acid peels also have been shown to promote neocollagenesis and improve benign pigmented lesions (Kitzmiller et al., 2003; Chun et al., 2004).

The development of new formulations through advances in nanotechnology and drug delivery systems is expected to further increase the use of topical agents as well as cosmeceuticals. Microneedling with a dermaroller has also been used to enhance drug delivery by creating pores in the stratum corneum, promoting neocollagenesis through release of various growth factors during the micro-wound healing process (Hou et al., 2017). Similarly, microdermabrasion using aluminum oxide crystals has been shown to be effective in improving the drug delivery and promoting dermal collagen synthesis. Recently, there is increasing evidence that stem cell-derived exosomes can ameliorate aging-related changes including UV-induced DNA damage and ROS generation and MMP-1 expression in senescent fibroblasts, and promote the expression of ECM proteins (Oh et al., 2018; Gao et al., 2021). The autologous stromal vascular fraction extracted from adipose tissue-derived stem cells has also shown to be effective in dermal rejuvenation due to its regenerative capacity (Charles-de-Sa et al., 2015; Rigotti et al., 2016). Still, clinical data are still insufficient, and further studies are needed to elucidate the anti-aging effects of exosomes and the stromal vascular fraction.

### 3.4 Injectables

The use of injectables in the dermatologic field has been increasing to improve rhytides and restore the soft tissue volume in aged skin. HA is one of the most commonly used injectables available due to its biocompatibility, ease of use, and reversibility. The injection of HA causes the dermis to stretch mechanically and enhances the structural support of the ECM, which activates dermal fibroblasts and leads to the production of type I collagen by activating the TGF-β signaling pathway (Wang et al., 2007; Turlier et al., 2013; Landau and Fagien, 2015). In addition, HA directly activates fibroblasts through its hyaluronan receptors, CD44 and CD168, causing them to migrate and proliferate (Mast et al., 1993; Turley et al., 2002). HA injection is also effective in improving skin hydration and texture (Ayatollahi et al., 2020). Recently, a novel EGF-containing HA filler was shown to induce types I and III collagen production and downregulate the expression of MMP-9 (Shin et al., 2022). In addition to HA, biocompatible polymers such as poly-L-lactic acid, polycaprolactone, and polynucleotide have also been found to stimulate fibroblasts and induce neocollagenesis and are thus increasingly used as injectables (Park et al., 2016; Kim J. H. et al., 2020; Oh et al., 2021). Furthermore, botulinum toxin injection not only overcomes hyperkinetic rhytides but also improves skin elasticity, skin hydration level and decrease skin erythema via suppression of neurogenic inflammation (Gazerani et al., 2009; Zhu et al., 2017). Table 2 summarizes the mechanisms of skin aging and corresponding dermatological interventions.

**TABLE 2:** Mechanisms of skin aging and dermatologic interventions to counteract them.

Clinical properties	Histological/Molecular changes	Strategy
Roughness		• Microdermabrasion
	SC compaction ↑	• Superficial chemical peels
	Epidermal thickness ↓	• Microneedling, fractional lasers, FRFM
	Epidermal HA ↓	• AHA, RA, EGF, peptides, estrogen, biopolymers (PDRN, PN)
		• Moisturization, LMWHA, acetylated HA
Solar lentigines		• TCA peels
	Elongation of RRs	• Lasers (CO_2_, Er:YAG, ablative fractional laser)
	Mutations of KC/MC genes	• FRFM
	MC No. ↑, melanogenesis ↑	• Selective photothermolysis
		• IPL, low-fluence Q-switched Nd:YAG laser
Wrinkles	Epidermal thickness ↓	• Microdermabrasion, superficial chemical peels, RA
		• Microneedling, fractional lasers, FRFM
	ROS → Inflammatory cytokines ↑	• Antioxidants, exosomes, GFs, PRP, SVF
	→ MMPs ↑	• Biopolymers (PDRN, PN)
	→ Degradation of ECM proteins	• Lasers, IPL, RF, HFUS
		• Synthetic polymers (PLA, PCL, PDO)
	More dermal changes in photoaged skin	• Photoprotection
Sagging	ROS → MMPs ↑	• Antioxidants, biopolymers, synthetic polymers, GFs, HFUS
		• Fibroblast stimulation by EBDs
	Elastic fiber degeneration	• Biopolymers, synthetic polymers, PRP, SVF
	Collagen degradation	• Fat injection, fillers
	Remaining disorganized elastic fibers	• Ablative fractional lasers, FRFM
Inelasticity	Solar elastosis	• Repeated chemical peels, topical RA
		• Ablative fractional lasers, FRFM
	Neutrophil elastase ↑	• Antioxidants
Edema	Vascular leakage ↑	• Massage and drainage (mechanical, US, RF, shock wave, acoustic wave)
	→ Fluid retention	
	Intervascular distance ↑ (loosely woven collagen network)	• Fibroblast stimulation (EBDs, injectables)
	Ectatic vessels with atrophic walls	• Selective photothermolysis, FRFM
Telangiectasia	Collagen and elastic fibers ↓	• Fibroblast stimulation (EBDs, injectables)
Redness	Perivascular inflammation	• Antioxidants, PRP, exosomes, PDRN, PN
Purpura	Neurogenic inflammation	• HFUS
		• Botulinum toxin, mild cryotherapy

Abbreviations: AHA, alpha-hydroxy acid; EBD, energy-based device; ECM, extracellular matrix; EGF, epidermal growth factor; FRFM, fractional radiofrequency microneedling; GF, growth factor; HA, hyaluronic acid; HFUS, high-frequency ultrasound; IL, interleukin; IPL, intense pulsed light; KC, keratinocyte; LMWHA, low-molecular weight hyaluronic acid; MC, melanocyte; MMP, matrix metalloproteinase; PCL, polycaprolactone; PDO, polydioxanone; PDRN, polydeoxyribonucleotide; PLA, poly (lactic acid); PN, polynucleotide; PRP, platelet-rich plasma; RA, retinoic acid; RF, radiofrequency; ROS, reactive oxygen species; RR, rete ridge; SC, stratum corneum; SVF, stromal vascular fraction; TCA, trichloroacetic acid; TNF, tumor necrosis factor; US, ultrasound.

### 3.5 Future perspectives

In very recent decades, researchers have attempted to counteract aging using senotherapeutics that selectively target senescent cells. Senotherapeutics are categorized into two groups. Senolytic drugs selectively eliminate senescent cells, and senomorphic drugs inhibit the negative effects of their SASPs. Since the combination of dasatinib and quercetin was proposed as the first senolytic drug to suppress genes that are increased in senescent cells, many studies have shown that various substances such as ABT-737, ABT-263, A1155463, and fiestin have anti-aging properties (Zhu et al., 2015; Thompson et al., 2022).

In particular, ABT-263 and ABT-737, which are Bcl-2 inhibitors, have been found to selectively eliminate SA β-gal-positive senescent cells in skin both *in vitro* and *ex vivo* (Victorelli et al., 2019; Kim et al., 2022a; Kim et al., 2022b; Park et al., 2022). Kim and his colleagues demonstrated that either ABT-263 or ABT-737 treatment selectively eliminated dermal fibroblasts in an intrinsic skin aging mouse model (Kim et al., 2022a). They also showed that the treatment increased the collagen density, epidermal thickness, and keratinocyte proliferation while reducing SASPs including MMP-1 and IL-6. After, this team revealed that treatment with ABT-263 and ABT-737 also attenuated the induction of MMPs and decreased collagen density in the photoaging mouse model (Kim et al., 2022b). In addition, ABT-263 showed potential in reducing pigmentation caused by photoaging in human skin inducing apoptosis of p16INK4A-positive fibroblasts with its senolytic activity, resulting in decreased levels of melanin and tyrosinase activity (Park et al., 2022).

One of the most notable targets of senomorphic agents is the mechanistic/mammalian target of rapamycin (mTOR) pathway, which regulates cellular metabolism and is linked to cellular growth, proliferation, and autophagy (Papadopoli et al., 2019). The mTOR pathway is also involved in the synthesis of SASPs (Cayo et al., 2021). Rapamycin, an mTOR inhibitor, exhibited significant reduction in senescence markers and SASPs as well as oxidative cellular stress in UV-induced photoaged human dermal fibroblasts (Bai et al., 2021). Moreover, Chung et al. revealed the potential anti-aging effect of topical application of rapamycin (an mTOR inhibitor) (Chung et al., 2019). A total of 17 subjects over the age of 40 years with age-related photoaging of the skin applied a rapamycin-containing hand cream to the dorsum of one hand and a placebo hand cream to the other hand daily for 8 months and found that the rapamycin-treated hand had a decrease in p16 and an increase in collagen VII protein.

Taken together, these promising results suggest that senotherapeutics may be a novel therapeutic option for skin aging; however, the limitations of these drugs, such as their specificity, selectivity, and efficiency, still need to be addressed, and their mechanisms of action and side effects must be better understood.

## 4 Conclusion

In conclusion, skin aging is a complex process that involves numerous biological and biochemical changes, and clinicians must have a thorough comprehension of skin aging physiology to devise an effective treatment plan. It is hoped that this narrative review will aid medical professionals in developing treatment plans to combat aging and gain a more complete understanding of the intricate process of skin aging.
